# TERT promoter C250T mutation in a recurrent cervical chordoid meningioma: a unique case report

**DOI:** 10.1186/s12957-025-04003-w

**Published:** 2025-09-26

**Authors:** Xuanbo Shao, Zhuofan Xu, Penghao Liu, Teng Zhang, Yang Feng, Shaxi Zhu, Zan Chen, Wanru Duan

**Affiliations:** 1 Department of Neurosurgery, Xuanwu Hospital, Capital Medical University, 45 Changchun Street, Xicheng District, Beijing, 100053 China; 2 Department of Orthopaedics and Traumatology, Medicine School, The University of Hong Kong, Hong Kong, China

**Keywords:** Meningioma, Chordoid, TERT, Spine

## Abstract

Chordoid meningioma (CM) is an uncommon histological variant of meningioma, typically found intracranially. Intraspinal occurrences, particularly in the cervical region, are exceedingly rare. We report a unique case of a 73-year-old male with cervical spinal CM who experienced tumor recurrence two years after gross total resection (GTR). Histopathological analysis confirmed the recurrence as anaplastic meningioma with telomerase reverse transcriptase (TERT) promoter C250T mutation, CNS WHO Grade 3—marking the first documented association of this mutation with spinal CM. This case underscores the diagnostic, molecular, and prognostic complexities of spinal CM and highlights the critical role of TERT promoter mutation in tumor behavior and classification.

**Supplementary Information:**

The online version contains supplementary material available at 10.1186/s12957-025-04003-w.

## Introduction

Meningiomas are the most common primary central nervous system (CNS) tumors in adults, typically originating from arachnoid cap cells [1]. Although the majority of meningiomas are regarded as benign (WHO grade 1) [2], certain histological subtypes, such as chordoid, clear cell, and anaplastic meningiomas, exhibit more aggressive behavior and are classified as WHO grades 2 or 3 [3]. CM, a rare variant, accounts for less than 1% of all meningiomas [4–6]. Histologically, it is characterized by cords or nests of epithelioid or vacuolated cells within a myxoid stroma [7].

CM is predominantly located in intracranial sites, and intraspinal occurrences are extremely rare. To date, only 14 cases of intraspinal CM have been reported in the literature (Table 1). In recent years, TERT promoter mutations have been identified as crucial molecular markers associated with tumor recurrence and poor prognosis, especially in higher-grade meningiomas [8, 9]. Consequently, the 2021 WHO Classification of Tumors of the CNS stipulates that meningiomas with a TERT promoter mutation should be classified as WHO grade 3.

**Table 1:** Summary of reported patients with spinal chordoid meningiomas

Author, year	No.of case	Age/Sex	Tumor location	Clinical symptoms	Duration symptoms(months)	Surgery	RT/CT	Recurrence	Recurrence Time for 1st recurrence(months)	Follow-up available(months)
Couce et al., 2000 [5]	1	28/F	C2	NA	NA	GTR	NO	NO	-	60
Ibrahim et al., 2005 [10]	1	26/M	C2-C3	Cramping and weakness in lower limbs	NA	GTR	NO	NO	-	2
Wu Liang et al., 2015 [11]	1	12/F	C2-C3	Numbness and weakness of right-side limbs and intermittent pain in the neck and right shoulder	3	PR	RT	YES	60	65
Yang Yang et al., 2016 [12]	5	NA	NA	NA	NA	NA	NA	NA	NA	NA
Sadashiva, et al., 2018 [13]	3	25/M	C3-C5	Quadriparesis	0.5	GTR	NO	NO	-	128
		36/F	Foramen Magnum to C3	Quadriparesis	0.5	GTR	NO	NO	-	162
		27/M	C1-C2	Neck pain and restriction of neck movements	8	GTR	NO	NO	-	40
Sugur, et al., 2018 [14]	2	NA	NA	NA	NA	NA	NA	NA	NA	NA
Tulloch, et al., 2020 [15]	1	45/F	C7-T5	Mid-thoracic back pain with bilateral leg weakness and sensory disturbances	5	GTR	NO	NO	-	24
Present case	1	71/M	C1-C2	Quadriparesis	2	GTR	NO	YES	24	31

*M* Male, *F* Female, *GTR* Gross total removal, *NA* Not available, *PR* Partial removal, *RT* Radiotherapy, *CT* Chemotherapy

**Fig. 1:**
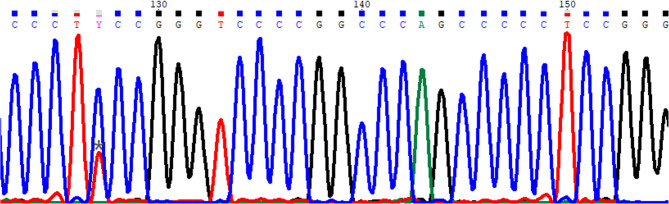
Sanger sequencing analysis showing a TERT promoter C250T mutation identified in the recurrent tumor specimen

This case report details a rare case of cervical CM that recurred as anaplastic meningioma with a TERT mutation, representing the first reported instance of an association between spinal CM and this genetic alteration (Fig. 1).

## Case presentation

A 73-year-old male presented to the outpatient clinic with a two-month history of limping and a recent two-week onset of progressive weakness in both upper limbs. There were no complaints of bowel or bladder dysfunction. Neurological examination revealed increased muscle tone and deep tendon reflexes in the right lower extremity. Muscle strength was graded as 4/5 in both the right upper and right lower limbs based on the Medical Research Council (MRC) scale. Sensory disturbances, including numbness in both hands and feet, were also reported, more pronounced on the right side. A positive Babinski sign was observed on the right. Laboratory evaluations, including routine blood and biochemical tests, were within normal limits.

**Fig. 2:**
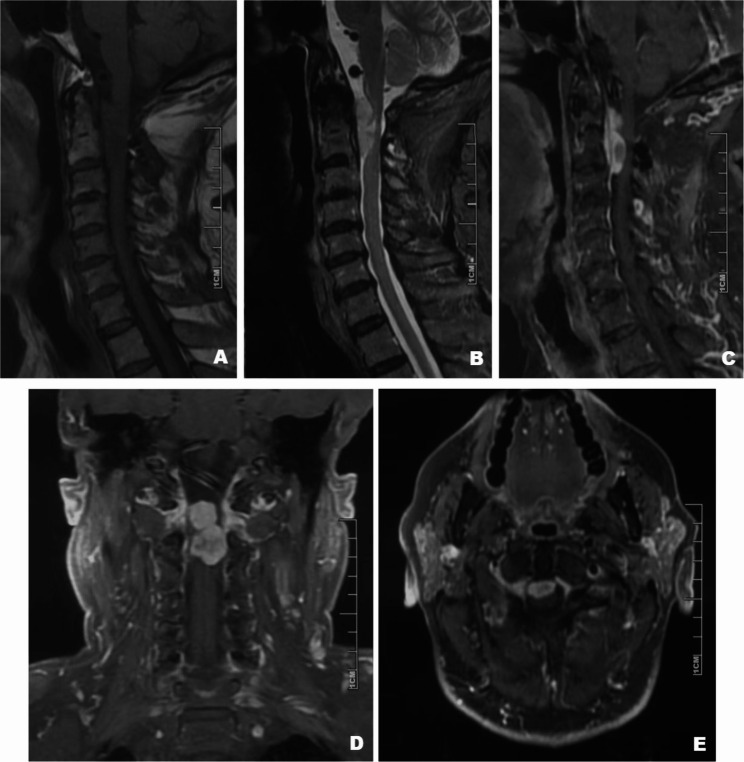
Preoperative MRI of the cervical spine demonstrating an extramedullary lesion at the C1–2 level. **A** Sagittal T1-weighted image shows isointensity; **B** Sagittal T2-weighted image reveals mixed hyperintensity; **C**–**E** Gadolinium-enhanced sagittal (**C**), coronal (**D**), and axial (**E**) T1-weighted images show heterogeneous contrast enhancement of the tumor

The patient had undergone prior spinal surgery two years ago for an intraspinal lesion at the C1–C2 level. At that time, pathological examination confirmed a diagnosis of meningioma, WHO grade 2, though the specific subtype could not be definitively identified, classifying it as a challenging case. Preoperative magnetic resonance imaging (MRI) of the cervical spine demonstrated a recurrent extramedullary lesion at the C1–C2 level, measuring 30 × 28 × 5 mm (Fig. 2). Based on the imaging characteristics, anatomical location, and surgical history, a diagnosis of recurrent meningioma was made, and the patient underwent surgical resection.

In order to validate the diagnosis, the original tumor specimen obtained during the patient’s initial surgery at an outside institution two years earlier was retrieved and re-examined.

**Fig. 3:**
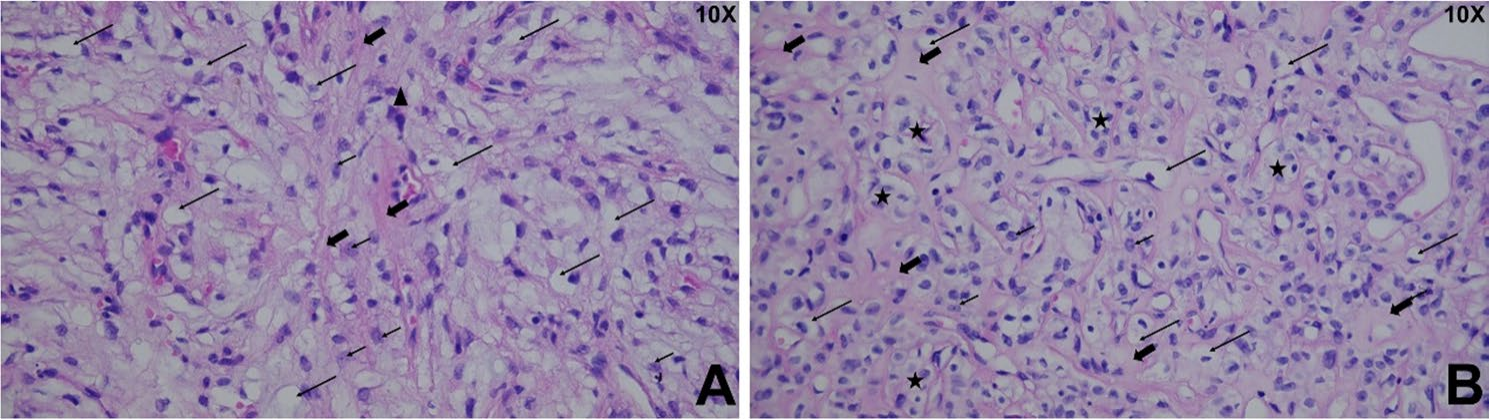
Hematoxylin and eosin (HE) staining of the tumor. **A** Tumor cells are ovoid and arranged in nests, surrounded by mucinous stroma (bold arrow). Cytoplasmic vacuoles (long arrow) and nucleoli (short arrow) are evident. **B** Tumor cells display mild atypia with abundant cytoplasm, visible vacuoles, and prominent nucleoli

Histopathological examination demonstrated tumor cells arranged in cords and nests within an abundant mucinous stroma (Fig. 3). The cells exhibited either clear or eosinophilic cytoplasm, with some showing prominent cytoplasmic vacuolization. Nuclear pleomorphism with fine chromatin and occasional nucleoli was noted. Swirl-like architectural patterns were present in certain areas.

**Fig. 4:**
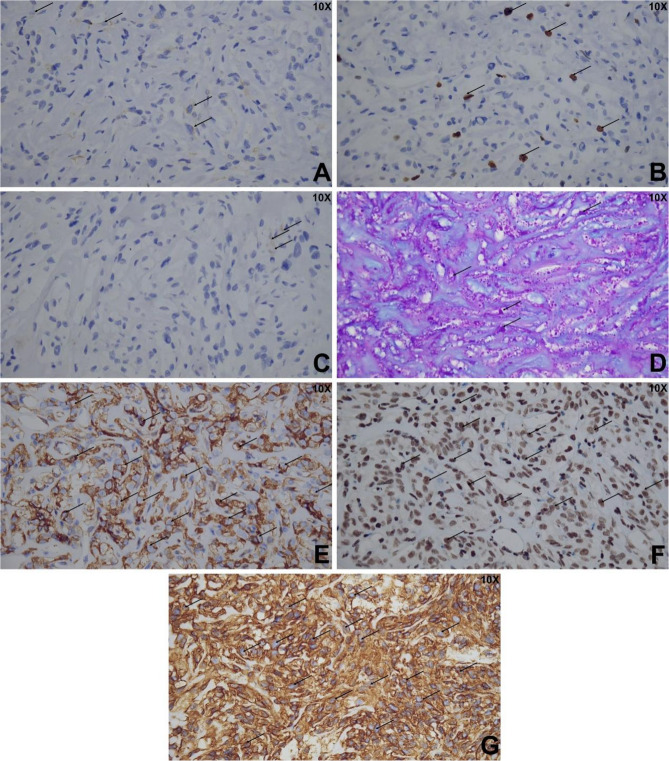
Immunohistochemical staining of tumor cells. **A** Weakly EMA-positive cells; **B** Ki-67-positive nuclei indicating a proliferation index of approximately 5%; **C** Weakly PR-positive cells; **D** PAS staining reveals cytoplasmic glycogen; **E** Diffuse expression of SSTR2; **F** SMARCE1-positive staining; **G** Vimentin-positive tumor cells

Immunohistochemical analysis revealed weak positivity for epithelial membrane antigen (EMA) in some tumor cells, with diffuse expression of somatostatin receptor 2 (SSTR2) and vimentin (Fig. 4). A small number of tumor cells were weakly positive for progesterone receptor (PR), while staining for desmin, S-100, and myogenin was negative. Periodic acid–Schiff (PAS) staining showed positive cytoplasmic glycogen deposits. There was no loss of nuclear expression for SMARCE1. The proliferative index, measured by Ki-67, was approximately 5%. Based on the above findings, we conclude that the original tumor should be diagnosed as a WHO grade 2 chordoid meningioma. For the recurrent tumor in this episode, molecular testing revealed the presence of a TERT promoter C250T mutation, a finding confirmed by Sanger sequencing. Thus, the final diagnosis of the recurrent lesion is anaplastic meningioma, CNS WHO grade 3.

**Fig. 5:**
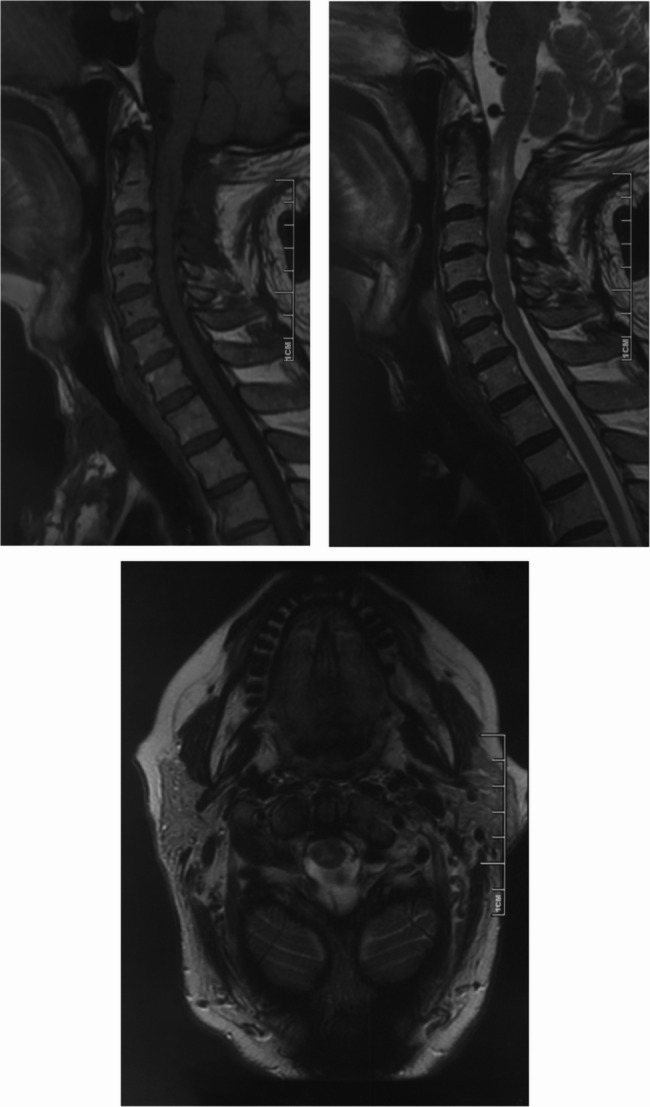
Postoperative MRI showing no evidence of tumor recurrence at the 7-month follow-up

After the surgical resection, the patient was transferred to the intensive care unit (ICU) due to poor respiratory function, underwent tracheotomy, and received ventilator-assisted ventilation. No adjuvant therapy was administered. Currently, the patient is still in the recovery phase, and his respiratory function is gradually improving. At 7 months post-surgery (Fig. 5), the patient demonstrated no signs of tumor recurrence on MRI.

## Discussion

CM, a rare histological subtype of meningioma, arises from meningothelial (arachnoid cap) cells [1]. More than 420 cases have been documented in the literature, with the majority located intracranially, especially in the supratentorial region [8]. Intraspinal CM is exceedingly rare. Since its first report by Couce et al. [5] in 2000, only 14 cases of spinal CM have been documented in the English literature [10–15]. The majority were located in the cervical region (especially C1–C3), with most patients under 40 years of age. This anatomical and demographic discrepancy from typical spinal meningiomas—which commonly occur in the thoracic spine and affect middle-aged individuals—suggests that spinal CM may represent a distinct clinical and pathological entity.

Clinically, spinal chordoid meningiomas typically present with limb weakness, sensory disturbances (such as numbness or pain), and limited cervical mobility. Symptom durations in reported cases range from several days to several months, with a mean course of approximately six months. In contrast, this patient experienced symptom onset only two months prior to presentation, which may suggest a more aggressive biological behavior. At 73 years of age, he represents the oldest reported case of spinal CM to date. Moreover, this is the only known case of recurrence following GTR, with tumor regrowth occurring within 24 months. These findings raise the possibility that advanced age may be associated with an increased risk of postoperative recurrence, underscoring the need for close long-term follow-up in elderly CM patients.

MRI remains the primary imaging modality for preoperative evaluation of spinal tumors. Yet, due to the lack of highly specific imaging characteristics, chordoid meningiomas often radiologically mimic conventional spinal meningiomas. In this case, non-specific imaging findings necessitated histopathological and immunohistochemical analyses to confirm the diagnosis and rule out other intraspinal lesions.

Histologically, chordoid meningiomas are characterized by epithelioid tumor cells arranged in cord-like or nest-like patterns embedded within a rich myxoid stroma. These tumors typically demonstrate diffuse immunopositivity for epithelial membrane antigen (EMA) and vimentin. In the present case, the morphological features were consistent with CM; however, the immunophenotype was atypical. Tumor cells exhibited weak EMA expression and focal positivity for PR, with strong, diffuse staining for SSTR2 and vimentin. PAS staining revealed glycogen accumulation within the cytoplasm. There was no loss of nuclear expression for SMARCE1, and the Ki-67 proliferation index was approximately 5%. Based on these findings, differential diagnosis included chordoma, chondrosarcoma, choroid glioma, myxopapillary ependymoma, and clear cell meningioma. However, the overall histomorphologic and immunohistochemical profile favored a diagnosis of chordoid meningioma.

In recent years, molecular diagnostics have become increasingly essential for prognostic stratification in central nervous system tumors. TERT promoter mutations, in particular, have been identified as significant markers of aggressive behavior and poor clinical outcomes. These mutations occur in approximately 5.7% of WHO grade 2 meningiomas [8]. This case confirms that chordoid meningiomas can undergo malignant progression to CNS WHO grade 3 anaplastic meningiomas driven by TERT promoter mutations, challenging the traditional perception that chordoid meningiomas exhibit relatively indolent biological behavior. Since molecular testing was not performed during the initial surgery, it remains unclear whether this mutation was inherently present or acquired during tumor recurrence. Additionally, due to the scarcity of molecular testing data for this subtype, the overall incidence of TERT mutations in intraspinal chordoid meningiomas remains undefined. These findings emphasize the importance of clinical follow-up and risk stratification for chordoid meningiomas, even for initially low-grade tumors, close monitoring and proactive molecular testing should be implemented for cases with atypical features (e.g., weak EMA/PR expression in this case) or elderly patients, as their histological “chordoid” characteristics do not preclude subsequent aggressive behavior. Furthermore, as the first reported case of an intraspinal chordoid meningioma progressing to an anaplastic meningioma with TERT mutation, this finding reveals the biological diversity of this subtype. It suggests that intraspinal and intracranial cases may possess distinct molecular profiles, highlighting the urgent need for further research into their unique progression pathways.

Surgical resection remains the primary and most effective treatment for chordoid meningioma. Evidence from several institutional studies suggests that the extent of resection is the strongest predictor of long-term tumor control. In this case, the tumor was completely resected; however, recurrence occurred within two years, likely due to adverse prognostic factors such as advanced age and the presence of a TERT promoter mutation. Given these risk factors, serial magnetic resonance imaging every 6 to 12 months is recommended to monitor for recurrence.

## Conclusion

Spinal chordoid meningioma remains a rare and diagnostically challenging tumor subtype, with limited clinical data available to inform standardized management strategies. This case highlights several unique characteristics: advanced patient age, tumor recurrence after GTR, and the presence of a TERT promoter mutation—the first reported instance in spinal CM. These findings emphasize the necessity of integrating molecular diagnostics into the routine assessment of atypical or recurrent spinal meningiomas. Future studies with larger cohorts are needed to better characterize the biological behavior of spinal CM, define the prognostic value of molecular alterations, and optimize long-term surveillance and treatment strategies.

## Supplementary Information

Supplementary Material 1.

## Data Availability

No datasets were generated or analysed during the current study.
