# Supplementary material for: TERT promoter C250T mutation in a recurrent cervical chordoid meningioma: a unique case report
Source: World J Surg Oncol. 2025 Sep 26;23:344. doi: 10.1186/s12957-025-04003-w (PMC12466048; doi:10.1186/s12957-025-04003-w)
Supplement: Supplementary file 1 — Supplementary Material 1. [file 12957_2025_4003_MOESM1_ESM.doc]

**TERT Promoter C250T Mutation in a Recurrent Cervical Chordoid Meningioma: A Unique Case Report**

Xuanbo Shao^1,*^, Zhuofan Xu^1,*^, Penghao Liu^1^,Teng Zhang^2^, Yang Feng^1^,Shaxi Zhu^1^,Zan Chen^1^,Wanru Duan^1^

**^*^**Xuanbo Shao and Zhuofan Xu contributed equally to this study as co-first authors.

**Author dffiliations:**

^1^Department of Neurosurgery, Xuanwu Hospital, Capital Medical University, Beijing, China

^2^Department of Orthopaedics and Traumatology, Medicine School, The University of Hong Kong, Hong Kong, China

**Academic degrees:**

Xuanbo Shao: Postgraduate

Zhuofan Xu: M.D.

Penghao Liu: M.D.

Teng Zhang: Ph.D.

Yang Feng: Undergraduate

Shaxi Zhu: Postgraduate

Zan Chen: M.D. Ph.D.

Wanru Duan: M.D.

**Corresponding Author:** Wanru Duan Department of Neurosurgery, Xuanwu Hospital, Capital Medical University, 45 Changchun Street, Xicheng District, Beijing, 100053, China

Email: [duanwanru@xwhosp.org](mailto:duanwanru@xwhosp.org)

**Statements & Declarations**

**Funding**

This review was supported by the following funding sources:

1.National Key R&D Program of China, "Research on Prevention and Control of Common Diseases" (No. 2023YFC2509700), funded by the Ministry of Science & Technology of the People's Republic of China.

2.Beijing Natural Science Foundation-Haidian Original Innovation Joint Fund (No. L232141), supported by the Beijing Municipal Science & Technology Commission.

3.Research and Application of Clinical Characteristic Diagnosis and Treatment Program (No. Z221100007422019), supported by the Beijing Municipal Science & Technology Commission.

**Ethics declarations**

**Ethics approval and consent to participate**

All authors have confirmed that this work complies with the International Committee of Medical Journal Editors (ICMJE) and the Declaration of Helsinki.

**Consent for publication**

Written informed consent was obtained from the patient for permission for publication of his personal and clinical details along with non-identifying images to be published in this study.

**Competing Interests**

The authors have no relevant financial or non-financial interests to disclose.

**Author Contributions**

X.S. and Z.X. wrote the main manuscript text and prepared Table 1.

P.L., T.Z., Y.F. and S.Z. annotated Figures 1–4, including MRI imaging, histopathological staining, and molecular sequencing data.

Z.C. and W.D. supervised the study design, reviewed pathological and molecular interpretations, and critically revised the manuscript for intellectual content.

All authors approved the final version and agree to be accountable for its content.

**Corresponding author**

Correspondence to Wanru Duan

**ORCID:**

Author Penghao Liu: 0000-0002-9100-1580

Author Zan Chen: 0000-0002-0104-115X

Author Zhuofan Xu: 0000-0002-6481-1238

Author Wanru Duan: 0000-0003-4751-3666

Author Xuanbo Shao: N/A

Author Shaxi Zhu: N/A

Author Yang Feng: N/A

Author Teng Zhang: N/A

**Acknowledgments**

We would like to express our most sincere gratitude to Dr. Leiming Wang from the Department of Pathology, Xuanwu Hospital, Capital Medical University. Thank him for providing professional technical support for the preparation and staining of pathological specimens in this study.
